# Advances in Gene Delivery Systems

**DOI:** 10.1155/2015/610342

**Published:** 2015-03-30

**Authors:** Surendra Nimesh, Sabina Halappanavar, Nagendra K. Kaushik, Pradeep Kumar

**Affiliations:** ^1^Department of Biotechnology, School of Life Sciences, Central University of Rajasthan, Bandarsindri, N.H. 8, Tehsil Kishangarh, District Ajmer, Rajasthan 305817, India; ^2^Genomics and Nanotoxicology, Laboratory Mechanistic Studies Division, Health Canada, Ottawa, ON, Canada K1A 0K9; ^3^Department of Electro and Biophysics, Plasma Bioscience Research Centre, Kwangwoon University, Wolgye-Dong, Nowon-Gu, Seoul 139-701, Republic of Korea; ^4^Nucleic Acids Research Laboratory, CSIR-Institute of Genomics and Integrative Biology, Delhi 110007, India

Gene therapy can be defined as incorporation of genetic material, that is, DNA or RNA, in the cellular gene regulation system, either to correct the expression of a malfunctioning gene or to modulate the cellular functions through expression of the newly incorporated gene. Nucleic acids (NAs) to correct/block malfunctioning genes have great therapeutic potential; however, their application is limited by their low cellular uptake, short half-life* in vivo*, rapid clearance, and quick enzymatic degradation. To circumvent this issue and achieve efficient and targeted gene delivery, numerous vectors have been developed, broadly categorized as viral and nonviral vectors. The viral vectors owing to their naturally evolved transduction properties were initially proposed as potential carriers due to site specificity, but their large clinical application is hindered because of immunogenicity and pathogenicity. These limitations and the advances in the field of nanotechnology have given rise to the development of nanoparticle-DNA delivery systems. Various delivery systems have been developed, each with their own inherent advantages and disadvantages. Modifications of delivery systems, using molecules such as PEG to increase residence time, and the use of cell-targeting peptides have greatly contributed to the efficacy of these delivery systems. Despite the advancements in this research field, there are still challenges and questions to overcome. These include the fate of the nanomaterials* in vivo*, their long-term side-effects on gene expression and toxicity as well as the conventional methods of ADMETox. Properly designed preclinical and clinical studies need to be undertaken to ensure the development of efficient and safe nanoparticle-DNA formulations. The need for more fundamental research into the understanding of gene function and gene delivery mechanisms inherently limits the development of successful nanoparticle-DNA formulations and so must also continue to evolve.

The discovery of gene therapy with potential advantages over existing biochemical technologies has proven to be an indispensable tool for elucidating molecular pathways and phenotype/genotype relationships. Because of various limitations attached to the stability of DNA in biological milieu, several parameters are to be taken care of, for the therapeutic success of gene therapy, such as (i) DNA protection, (ii) high transfection efficacy, (iii) reduced toxicity and absence of nonspecific effects, (iv) high potency even at dosage of DNAs, (v) adaptation to various treatment regimens as well as diseases, and (vi) efficient vectors to bypass intracellular and extracellular barriers to reach their target tissue/organ.

In spite of much research being carried out with the aim to design different types of vectors for DNA delivery, safe and efficient delivery of DNA into target cells or organs still remains a big challenge. The DNA delivery is a multistep process where a series of extra- and intracellular barriers have to be bypassed for successful application and could be achieved with efficient vectors. For a vector to be an efficient delivery vehicle, it should consist of three different functional moieties: (i) a cationic polymer or lipid component to condense DNA and form complexes, which facilitates their endosomal escape after cellular uptake and facilitates effective unpacking of the complexes in the cytoplasmic compartment; (ii) a hydrophilic component, such as PEG, to impart solubility and stability to the complexes in the biological environment; (iii) a ligand that is specific for the target cell/tissue for enhanced target ability to impart more efficient cellular uptake by receptor-mediated endocytosis. Further, differences in angiogenesis and metastasis between cancerous and normal cells can be exploited to engineer nanoparticle-DNA complexes to be employed to target tumour cells and, henceforth, for designing target-specific vectors. With nanoparticle-DNA complexes gaining successful applications, polymeric nanoparticles are rapidly emerging as DNA delivery systems both* in vitro* and* in vivo*. A review of the literature suggests that polymeric nanoparticles can be used to deliver functional DNA to the target cells. Various tumour models have also been used to conduct studies with nanoparticles to deliver DNA for antitumour treatments. A large number of successful animal model studies, including systemic delivery to nonhuman primates, provide excitement and enthusiasm for upcoming research in the field but still a number of hurdles and concerns must be overridden before DNA delivery could be harnessed as a new therapeutic modality.

For nanoparticles to be designated as an ideal DNA carrier system, the nanoparticles must possess long circulation time, low immunogenicity, good biocompatibility, selective targeting, and efficient penetration to barriers such as the vascular endothelium and the blood brain barrier, self-regulating release without clinical side effects. The successful implication of DNA complexes in clinical applications requires exhaustive details pertaining to efficacy and pharmacokinetics of DNA-vector systems. There is still a huge amount of effort required in preparation of better polymers and in the development of better ways of encapsulating or complexing DNA with them. In this era of developing genetic therapeutics, there is a call for the collaborative work by academicians and industry groups in order to develop methods for preparation of more stable polymeric nanoparticles/DNA complexes and robust analytical methods to characterize formulations during formation and storage.

*Surendra Nimesh* *Sabina Halappanavar* *Nagendra K. Kaushik* *Pradeep Kumar*

